# A Severe Case of Ludwig’s Angina with a Complicated Clinical Course

**DOI:** 10.7759/cureus.7695

**Published:** 2020-04-16

**Authors:** Vitaley Kovalev

**Affiliations:** 1 Acute Care Surgery, California Hospital Medical Center, Los Angeles, USA; 2 Basic Medical Sciences, College of Osteopathic Medicine of the Pacific, Western University of Health Sciences, Pomona, USA

**Keywords:** ludwig's angina, necrotizing fasciitis, odontogenic infection, surgery, tracheostomy

## Abstract

Ludwig's angina is a cellulitis of the submandibular, sublingual, and submental spaces, which tends to spread rapidly along fascial planes. The most common cause is a dental infection, although any other oropharyngeal infection has the potential to develop into Ludwig’s angina. The most feared complication of Ludwig’s angina is airway obstruction. Treatment involves early recognition so that an airway can be secured, initiation of antibiotics, and, finally, potential surgical debridement. We describe the case of a 57-year-old male with multiple comorbidities who was seen by a provider three times for dental pain prior to his admission for Ludwig's angina. Upon his index admission, he was found to have Ludwig’s angina with impending airway obstruction. He required an emergency surgical airway debridement and extraction of multiple teeth. Although the patient eventually recovered, his hospital stay was prolonged and marked by multiple complications. This case is an example of a severe presentation of Ludwig's angina and the difficulties faced by the medical team in managing this condition. Early recognition and rapid intervention are paramount in the management of this serious condition.

## Introduction

Ludwig's angina is a potentially life-threatening cellulitis of the floor of the mouth and was first described by the German army physician Wilhelm Frederick von Ludwig in 1836 [1]. It involves the submandibular, sublingual and, submental spaces and tends to spread rapidly along fascial planes. Patients who are more commonly affected tend to be male, in the fourth decade of life, and from a lower socioeconomic background [2,3]. The most common condition that causes Ludwig’s angina is an odontogenic infection. Other potential causes include peritonsillar abscess, mandibular fracture, penetrating injury to the floor of the mouth, mandibular osteomyelitis, otitis media, tongue piercing, and sialolithiasis of the submandibular glands [4,5]. Comorbidities that are associated with the development of Ludwig's angina are diabetes mellitus, hypertension, and immunocompromised status [2,6]. Most infections are polymicrobial and include anaerobic and aerobic bacteria. The most feared complication of Ludwig’s angina is airway obstruction. Other complications include mediastinitis, carotid artery rupture, internal jugular vein thrombophlebitis, empyema, necrotizing fasciitis, osteomyelitis, and aspiration pneumonia [7]. The reported mortality rates range from 0.3% to 11.8% [6,8]. Patients usually present with fever, pain, and swelling of the floor of the mouth. Timely recognition and initiation of treatment are paramount because the infection can progress rapidly. Treatment includes antibiotics, corticosteroids, securement of the airway, and surgical debridement and drainage [2]. This article presents the case of a 57-year-old male with multiple comorbidities who developed a severe form of Ludwig’s angina due to an odontogenic infection.

## Case presentation

The patient is a 57-year-old male with a medical history of chronic obstructive pulmonary disease, congestive heart failure, hypertension, morbid obesity, and stage III breast cancer with the last chemotherapy completed three weeks before the index admission. He initially presented to our Emergency Department (ED) with left lower dental pain for four days. Before that visit, he had already seen his primary care physician three days ago and had received a prescription for amoxicillin-clavulanate 875 mg/125 mg twice per day for 10 days and ibuprofen for a presumed dental infection, which the patient has been taking. On the initial visit, his vital signs were normal, and he was afebrile with multiple missing and fractured upper and lower molars on the left side with mild gingival edema of the left lower mandible. His white blood cell count was 9.4 x 10^3^/μL. A computed tomography (CT) scan with intravenous (IV) contrast of the neck was obtained, which revealed periapical abscesses of tooth numbers 8, 9, 10, 17, and 19, with mild adjacent soft tissue swelling. He also had fractures of teeth number 8 through 10. The patient was asked to continue amoxicillin-clavulanate and follow-up with his dentist, with whom he already had a scheduled appointment in two weeks. Two days later, the patient returned to the ED for the same dental pain. Because there was no change in his physical examination, no new labs were ordered, and the patient was prescribed clindamycin 300 mg three times per day for a 10-day course and again asked to follow-up with his dentist.

**Figure 1:**
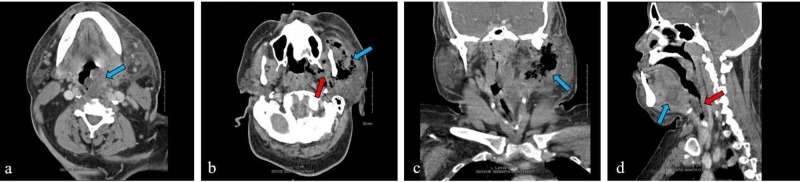
Contrast-enhanced computed tomography of the soft tissues of the neck showed rightward displacement of the hypopharyngeal airway (a, blue arrow, axial image). Also noted were abscesses of the left masticator muscle (b, blue arrow, axial image), medial and lateral pterygoids (b, red arrow, axial image), left stylomandibular canal (c, blue arrow, coronal image), and the sublingual space (d, blue arrow, sagittal image) with narrowing of airway to 4 mm (d, red arrow, sagittal image).

In three days, the patient once again returned to the ED with worsening dental pain and now facial swelling. His blood pressure was 146/72 mm Hg, heart rate was 86/minute, respiratory rate was 18/minute, temperature was 36.7°C, and oxygen saturation was 99%. On examination, he had difficulty in controlling oral secretions and also had a muffled voice, stridor, and swelling on the left side of the face at the mandibular angle with tenderness and crepitus on palpation. There was gingival swelling of the left lower mandible and trismus, but no swelling or tenderness of the floor of the mouth was reported. His laboratory results revealed a leukocytosis of 18.7 x 10^3^/μL with 90.2% neutrophils and creatinine 1.4 mg/dL from a baseline of 1.0 mg/dL. A repeat CT scan of the neck with IV contrast was obtained (Figure 1). The patient was started on piperacillin-tazobactam, vancomycin, clindamycin, dexamethasone, and 500-mL bolus of crystalloid. The intensivist and general surgery were immediately consulted because of the concern of an impending airway compromise. The initial plan was to transport the patient directly to the operating room for a controlled intubation. By the time the patient arrived at the operating room, he had had difficulty in phonating and controlling his airway. Rapid sequence intubation was attempted in a lateral position by anesthesia with video laryngoscopy, but the endotracheal tube could not be passed through the pharynx due to severe edema. The general surgeon then attempted an emergency cricothyroidotomy, but again, because of severe edema of the neck, he was not able to pass the tube in two attempts. A tracheostomy was then attempted. The strap muscles were edematous but allowed for dissection and successful cannulation of the trachea. The patient was moved to the intensive care unit (ICU) in a stable condition for continued resuscitation. An Oral and maxillofacial surgeon was consulted. On hospital day 2, the oral and maxillofacial surgeon took the patient to the operating room for debridement of infected tissue. A surgical extraction of tooth numbers 17, 19, 21, and 22 was performed. The submandibular space and buccal space were entered, and a large volume of pus was evacuated. A 2.5-inch-long Penrose drain was placed in the submandibular space. The masticator and parapharyngeal spaces were reached by blunt dissection reflecting the lingual gingiva around the extraction socket of tooth number 17. The left masticator space was drained. Cultures were obtained, and a nasogastric tube was placed. On the third postoperative day, the cultures returned positive for Klebsiella pneumoniae, which was sensitive to piperacillin-tazobactam, Streptococcus viridans group, and coagulase-negative Staphylococcus aureus. The antibiotics were then consolidated to piperacillin-tazobactam. The patient passed the swallow evaluation on postoperative day 7 and was started on pureed foods. However, when the speech pathologist attempted a flexible laryngoscopy to evaluate swallowing, an unexpected nasopharyngeal mass was found, which precluded the passage of the laryngoscope. An otolaryngologist was consulted and the patient was taken to the operating room for mass excision and biopsy on hospital day 16. A pedunculated, lobular mass was found on the roof of the nasopharynx that was blocking half of the nasopharynx. The mass was excised and sent to pathology. The patient was transferred in a stable condition to the medical-surgical floor. The pathology report described the mass as hypertrophied benign lymphoid tissue. Two days after the nasopharyngeal mass excision, the patient began having intermittent minor bleeding from the mouth, as noted by the nursing staff. His aspirin and unfractionated heparin doses were stopped. On the third day after the mass excision, the patient began to have profuse oral bleeding and therefore rapid response was called. The otolaryngologist was also called, and the suspected bleeding from the biopsy site and the nasopharynx was packed at bedside with a norepinephrine-soaked balloon pack. The patient was also noted to have new swelling to the left side of the neck and was dyspneic. He was sedated, placed back on mechanical ventilation, and moved back to the ICU. The packing stopped the bleeding, but the source was not identified. Interventional radiology (IR) was consulted for possible embolization. That evening, on hospital day 19, the patient was taken to the IR suite. The radiologist discovered a large pseudoaneurysm of the left internal maxillary artery with active bleeding, which the radiologist was able to embolize, and no further bleeding was reported. Later, on hospital day 22, the patient suddenly developed atrial fibrillation with rapid ventricular response and was started on an amiodarone infusion, and a CT angiogram (CTA) of the chest was obtained. The CTA revealed a partially occlusive thrombus extending from the right main pulmonary artery into the lobar and segmental branches of the right lower and upper lobes. Anticoagulation at that point was contraindicated given a recent oral hemorrhage; therefore, on hospital day 24, an inferior vena cava filter was placed. The patient continued to recover and was discharged to a rehabilitation center on hospital day 31.

## Discussion

Ludwig’s angina is a potentially life-threatening cellulitis of the submandibular, sublingual, and submental spaces and tends to spread rapidly along fascial planes. Our patient developed Ludwig’s angina as a result of multiple dental abscesses as he was not able to see a dental surgeon soon enough to address the developing infection. Just as with our patient, most deep space neck infections are of odontogenic origin, especially the second and third molars [9]. Other reported sources of infection are sialadenitis, peritonsillar abscess, mandibular fracture, penetrating injury to the floor of the mouth, mandibular osteomyelitis, otitis media, tongue piercing, and sialolithiasis of the submandibular glands [4,5].

Most cases of Ludwig’s angina are polymicrobial and include gram-positive, gram-negative, and anaerobic bacteria. Commonly isolated organisms in order of descending frequency include Streptococcus viridans, Staphylococcus aureus, Neisseria species, Haemophilus species, Bacteroides species, non-A β-streptococcus, Streptococcus pyogenes, Fusobacterium, Prevotella, and Eikenella [9]. Our patient’s case is unusual in that K. pneumoniae was isolated in addition to the expected culprits.

Undoubtedly, our patient’s clinical course was complicated by his comorbidities, including recent chemotherapy. Immunocompromised patients with diabetes mellitus and HIV (human immunodeficiency virus) have been shown to have an increased risk of complications with more intense and longer hospital stays [6]. Additional conditions that have been associated with worse prognosis are poor dental hygiene, obesity, malnutrition, alcoholism, hypertension, and having a tracheostomy [6,10].

The most common presenting symptoms of Ludwig’s angina are oral and dental pain, dysphagia and odynophagia, sore throat, otalgia, respiratory distress, and change of voice [2,9]. On inspection, the patient may have neck swelling, trismus, halitosis, sialorrhea, gingival swelling, or muffled voice [2]. Another physical examination finding described in the literature is a “double tongue sign” that involves an elevation of the floor of the mouth caused by edema of the submandibular space [11]. On palpation, there may be cervical adenopathy and a characteristic “woody” induration of the floor of the mouth, which was not noted in our patient [12].

A timely diagnosis is imperative so that appropriate treatment can be started. The most feared complication of Ludwig’s angina is airway compromise. If the airway is not secured early enough, as in our patient, then intubation will be impossible, and an emergency surgical airway would be the only option. Findings from a retrospective review even advocated the use of an elective awake tracheostomy as a safer alternative than endotracheal intubation [13].

Antibiotics should be started as soon as the diagnosis is suspected. Commonly recommended initial regimens include penicillin G, metronidazole, and clindamycin [13,14]. Because of the severity of this infection, most providers will often opt for broader coverage. However, in a retrospective review of 42 patients admitted for Ludwig’s angina, the most common organisms were found to be susceptible to penicillin, and only 3 patients required a change of antibiotics [15]. Notably, our patient’s culture found K. pneumoniae, which was resistant to ampicillin but sensitive to piperacillin-tazobactam.

The addition of steroids has been suggested in the literature to allow for easier intubation and possibly avoiding a surgical airway. Steroids may also allow better penetration of antibiotics into the fascial space by reducing edema [16]. A course of dexamethasone 10 mg every eight hours for 48 hours has been suggested [14]. A narrative review of 17 articles investigating the utility and safety of steroids concluded that even though the role of steroids in Ludwig’s angina remains uncertain, there is no suggestion of adverse effects in the described cases, and steroids may be beneficial [17].

Surgical drainage and debridement are reserved for the severe cases of Ludwig’s angina or in cases in which there is no improvement with antibiotics and steroids. Surgical intervention involves decompressing the submental, submandibular, and sublingual spaces by external incision and drainage [13]. Source control is also important. Because most infections are odontogenic in origin, any dental infections or fractured teeth need to be addressed as well.

## Conclusions

Our case is unusual in its severity and is an example of the most serious presentation of Ludwig's angina. Ludwig's angina is a potentially life-threatening condition that can progress rapidly. The most feared complication is airway obstruction, which can make intubation impossible and may necessitate a surgical airway. The treatment involves early recognition, securement of the airway, initiation of antibiotics, and potential surgical drainage or debridement. In this paper, we presented a case of Ludwig's angina, which, due to an unfortunate delay in dental surgery referral, resulted in the development of its most severe form. The patient’s multiple comorbidities further complicated his clinical course and prolonged the recovery period.

## References

[1] Shemesh A., Yitzhak A., Ben Itzhak J., Azizi H., Solomonov M (2019). Ludwig angina after first aid treatment: possible etiologies and prevention-case report. J Endodon.

[2] Almutairi DM, Alqahtani RM, Alshareef N, Alghamdi YS, Al-Hakami HA, Algarni M (2020). Deep neck space infections: a retrospective study of 183 cases at a tertiary hospital. Cureus.

[3] Karkos PD, Leong SC, Beer H, Apostolidou MT, Panarese A (2007). Challenging airways in deep neck space infections. Am J Otolaryngol.

[4] Chow AW (2010). Infections of the oral cavity, neck and head. In: Principles and Practice of Infectious Diseases, 7th ed.

[5] Balasubramanian S, Elavenil P, Shanmugasundaram S, Himarani J, Krishnakumar Raja V (2014). Ludwig's angina: a case report and review of management. Res Dent Sci.

[6] Botha A, Jacobs F, Postma C (2015). Retrospective analysis of etiology and comorbid diseases associated with Ludwig’s angina. Ann Maxillofac Surg.

[7] Read-Fuller A, Mueller A, Finn R (2015). Maxillofacial infections. Sel Readings Oral Maxillofac Surg.

[8] McDonnough JA, Ladzekpo DA, Yi I, Bond WR Jr, Ortega G, Kalejaiye AO (2019). Epidemiology and resource utilization of ludwig’s angina ED visits in the United States 2006-2014. Laryngoscope.

[9] Prabhu SR, Nirmalkumar ES (2019). acute fascial space infections of the neck: 1034 cases in 17 years follow up. Ann Maxillofac Surg.

[10] Whitesides L, Cotto-Cumba C, Myers R.A (2000). Cervical necrotizing fasciitis of odontogenic origin: a case report and review of 12 cases. J Oral Maxillofac Surg.

[11] Watari T, Tokuda Y (2020). Double tongue signs in a case of submandibular space infection. BMJ Case Rep.

[12] Hurley M, Heran M (2007). Imaging studies for head and neck infections. Infect Dis Clin North Am.

[13] Parhiscar A, Har-El G (2001). Deep neck abscess: a retrospective review of 210 cases. Ann Otol Rhinol Laryngol.

[14] Lustig LR, Schindler JS (2018). Ear, nose, & throat disorders. Current Medical Diagnosis & Treatment.

[15] Varghese L, Mathews SS, Antony Jude Prakash J, Rupa V (2018). Deep head and neck infections: outcome following empirical therapy with early generation antibiotics. Trop Doct.

[16] Har-El G, Aroesty J, Shaha A, Lucente F (1994). Changing trends in deep neck abscess: a retrospective study of 110 patients. Oral Surg Oral Med Oral Pathol.

[17] Tami A, Othman S, Sudhakar A, McKinnon BJ Ludwig's angina and steroid use: a narrative review. Am J Otolaryngol.

